# Occurrence of Multidrug-Resistant Candidiasis Among Pregnant Women at a Peri-Urban Hospital, Uganda

**DOI:** 10.7759/cureus.97603

**Published:** 2025-11-23

**Authors:** Lucas Ampaire, Deogratious Mukasa, Marvin Bikamatte, Winston Katatanzi, Emmanuel Mukula, Agnes P Akello, Aggrey Byaruhanga, Enoch Muwanguzi, Moses Ocan, Benson Okongo, Jazira Tumusiime, Charles Nkubi Bagenda

**Affiliations:** 1 Department of Medical Laboratory Science, Mbarara University of Science and Technology, Mbarara, UGA; 2 Laboratory Department, Mbale Police Health Centre III, Mbale, UGA; 3 Department of Microbiology, Our Lady of Consolata - Kisubi Hospital, Kisubi, UGA; 4 Department of Microbiology, Kiwoko Hospital, Kiwoko, UGA; 5 Department of Haematology, St. Charles Lwanga Buikwe Hospital, Buikwe, UGA; 6 Laboratory Department, Ogur Health Center IV, Ogur, UGA; 7 Department of Public Health, Ministry of Health, Kampala, UGA; 8 Department of Pharmacology and Therapeutics, Makerere University College of Health Sciences, Kampala, UGA

**Keywords:** antifungal resistance, candida albicans, candida krusei, candida tropicalis, pregnancy, vulvovaginal candidiasis

## Abstract

Introduction: Vulvovaginal candidiasis(VVC)is one of the most common non-bacterial infections in women worldwide. The common causative yeasts are *Candida albicans* and *Candida krusei*. Limited studies on antifungal resistance have been conducted in low-resource settings like Uganda. In this study, we aimed to establish the prevalence and drug susceptibility patterns of VVC among pregnant women at a peri-urban private not-for-profit referral hospital in Uganda.

Methods: A total of 352 vaginal discharge swabs were collected from consenting pregnant women attending the antenatal clinic of Kisubi Hospital, Uganda. Microscopy and culture on Sabouraud dextrose agar were used for primary screening. *Candida* species identification and differentiation were achieved using the germ tube tests and Candida Chromogenic Agar (Oxoid Ltd, Hampshire, United Kingdom). Antifungal susceptibility testing was performed using the standard Kirby-Bauer diffusion method as previously described. We included voriconazole (1 μg), fluconazole (100 μg), itraconazole (1 μg), nystatin (100 μg), caspofungin (5 μg), and interpreted as per the guidelines of Clinical Laboratory Standard Institute 2022. We used SPSS Statistics for Windows, version 15 (SPSS Inc., Chicago, Illinois, United States) for descriptive analysis of the data.

Results: *Candida* species were isolated in 93 (26.4%) pregnant women studied. These included *C. albicans* (n=51, 43.2%), *C. krusei* (n=46*, 39*.0%), *Candida tropicalis* (n=9, 7.6%), and unidentified *Candida* species (n=12, 10.2%). All *Candida* species showed resistance to at least one class of drug, with a total resistance of 86.9% and a multidrug resistance of 56.3%. *C. albicans* and *C. krusei* exhibited the most resistance up to 100% to azoles and caspofungin. All species exhibited the least resistance to nystatin at 76.3%.

Conclusion: *C. albicans* and *C. krusei* remain significant causes of candidiasis among pregnant women in our setting. The observed multidrug resistance in *C. albicans* poses a great threat to the management of candidiasis.

## Introduction

Vulvovaginal candidiasis (VVC) is the second most common cause of vaginitis worldwide, after bacterial infection. The risk of VVC for non-pregnant women is approximately 20%, but it increases by 30% during pregnancy [1]. Worldwide, recurrent VVC affects about 138 million women annually (range, 103-172 million), with a global annual prevalence of 3871 per 100,000 women; 372 million women are affected by recurrent VVC over their lifetime [2].

In sub-Saharan Africa, about one-third of the women have VVC caused by *Candida albicans* [3]. Osman Mohammed et al. declared that the prevalence of VVC in Eastern Africa is at 29.5%, North Africa at 15.3%, and West Africa at 29.9% [4]. There are an estimated 7,345,000 women in Uganda aged 15-45 years. There are 1,448,007 pregnancies per year, and an estimated 1,086,000 women are pregnant at any one time. In Uganda, recurrent vaginal candidiasis is estimated to occur in up to 651,600 pregnant women per year [5].

*C. albicans* has long been known to be the primary pathogen responsible for the infection; however, an epidemiological shift toward non-*C. albicans* (NCA) is now observed across the globe [6]. Other species such as *Candida glabrata*, *Candida krusei*, and *Candida parapsilosis* are emerging causative agents and in some clinical settings, in a higher proportion than *C. albicans*. Notably, these NCAs have been associated with recurrent maternal VVC. Alarmingly, co-infection of *C. albicans* and NCAs has also been reported [7]. 

*Candida* species exhibit varying degrees of susceptibility to the most commonly used antifungal agents. *C. krusei* is intrinsically resistant to fluconazole, with a global resistance rate of 78.3%. *C. glabrata* displays reduced dose-dependent susceptibility compared with other *Candida* species and has a global resistance rate of 15.7%. Primary resistance to fluconazole is rare for *C. albicans* (1.4%), *C. parapsilosis* (3.6%), and *Candida tropicalis* (4.1%) [8]. The management of the VVC in our setting largely remains empiric, and yet, there is a reported shift towards NCAs and the emergence of antifungal resistance. Additionally, due to limited laboratory infrastructure, there is scant data on aetiology, prevalence, and antifungal patterns of maternal *Candida* species in Uganda. In this study, we sought to determine the prevalence of VVC, its aetiology, and antifungal susceptibility patterns in the central region of Uganda.

## Materials and methods

Study design, setting, and population

This was a cross-sectional study done among pregnant women attending antenatal care at Our Lady of Consolata Kisubi Hospital, a peri-urban private not-for-profit referral hospital, in Kisubi, in the Central region of Uganda. All study procedures and their importance were explained to the participants in their local languages before recruitment. A total of 352 randomly recruited pregnant women participated in this study.

Ethical approval

The study protocol was reviewed and approved by the Faculty of Research Committee, Mbarara University of Science and Technology (approval number: MUST/MLS/030). Written informed consent was obtained from all the participants. Confidentiality and privacy of participants were upheld throughout the study procedures. All pregnant women confirmed with Candida colonizations were sent to the Clinical Team at the Maternal Child Health Department for further management.

Sample collection

A pair of high vaginal swab samples was collected by the midwife or gynecologist from each participant following the locally approved standard operating procedure. One of the swabs was put into a dry holder container, and the other into the holder container with Amies transport medium. The swabs were then uniquely labeled and transported at room temperature to the clinical microbiology laboratory immediately and processed within one hour of collection. 

Laboratory analysis

Microscopy

We used dry vaginal discharge-cotton swabs to observe for suspect yeast cells/morphotypes. It involved both observation of Gram-stained smears and wet preparation that were performed following the locally approved standard operating procedures by two laboratory technologists.

Fungal Culture, Identification, and Antifungal Susceptibility Testing 

Fungal culture was performed using the swabs collected in the Amies Transport media. The swabs were streaked on Sabouraud’s dextrose agar (Oxoid Ltd, Hampshire, United Kingdom) containing 2% chloramphenicol and cultured at 37°C for 48 hours. Occurrence of yellow cream mucoid colonies and microscopic observation of gram-positive stained ovoid yeast cells and pseudohyphae from the resultant colonies were regarded as suspect *Candida*. For quality control, a known positive *Candida* sample was also plated and cultured alongside the samples.

Fungal species identification was done using the germ tube test and growth on CHROMAgar^TM ^Candida (CHROMagar, Paris, France). The presence of sprouting yeast cells (tube-like outgrowth) confirmed the presence of *C. albicans* in the sample, and those without tube-like outgrowth were reported as NCA isolates. The NCAs were then identified using the colonial characteristics on ChromAgar^TM^ Candida.

Antifungal susceptibility testing using fluconazole (100 µg), Itraconazole (10 µg), voriconazole (10 µg), nystatin (100 IU), and caspofungin 5 μg (Bioanalyse Medical Materials Industry and Trade Anonim Company, Ankara, Türkiye) on cultures of *Candida* isolates was achieved using the Kirby-Bauer disc diffusion method and a 0.5 McFarland standard equivalent of inoculum. We used Mueller-Hinton agar (Oxoid Ltd) supplemented with glucose 2% (w/v). Inoculum suspensions of both clinical isolate and control *Candida* species (*C. krusei* ATCC 6258, *C. albicans* ATCC 10231, *C. tropicalis* ATCC 90030) were incubated at 37ºC for 24 hours. The diameters of zones of inhibition were measured in millimeters using a ruler. The results were interpreted according to the Clinical Laboratory Standard Institute, 2022 document [9]. Multi-drug resistance was considered as isolates being non-susceptible to at least one drug in each category of antifungal drugs used. 

Data analysis

Data were analyzed using SPSS Statistics for Windows, version 15 (SPSS Inc., Chicago, Illinois, United States). Categorical variables were summarized as frequencies and percentages. Comparisons between participants with and without VVC were performed using the Chi-square test or Fisher’s exact test to determine statistically significant differences. A p-value of less than 0.05 was considered statistically significant. The prevalence of VVC, along with its 95% confidence interval (CI), was calculated as a proportion of the total study population. The common *Candida *species causing VVC and their drug susceptibility patterns were expressed as frequencies and percentages. The results were presented using tables and figures.

## Results

Participant demographic characteristics

Among the 352 study participants, 93 (26.4%) had VVC, while 259 (73.6%) did not. Most participants were aged 21-29 years, married, and had secondary or tertiary education. VVC was slightly more common among women aged 30-39 years, married, employed, multiparous, and in the third trimester of pregnancy, though these differences were not statistically significant. Nearly all participants had one sexual partner, and about half reported a history of sexually transmitted disease. A previous history of *Candida* infection, antibiotic use, and chronic conditions was slightly more frequent among those with VVC, but none of these associations reached statistical significance (p > 0.05), as shown in Table 1.

**Table 1:** Characteristics of the study participants stratified by vulvovaginal candidiasis STD: sexually transmitted disease

Variable	Total (n=352), n (%)	Vulvovaginal candidiasis		p-value
		Absent (n=259), n (%)	Present (n=93), n (%)	
Age groups (years)				0.41
≤20	37 (10.5%)	29 (11.2%)	8 ( 8.6%)	
21-29	186 (52.8%)	138 (53.3%)	48 (51.6%)	
30-39	118 (33.5%)	82 (31.7%)	36 (38.7%)	
40-49	11 ( 3.1%)	10 ( 3.9%)	1 ( 1.1%)	
Marital status				0.071
Not married	59 (16.8%)	49 (18.9%)	10 (10.8%)	
Married	293 (83.2%)	210 (81.1%)	83 (89.2%)	
Education				0.45
None	3 ( 0.9%)	1 ( 0.4%)	2 ( 2.2%)	
Primary	63 (17.9%)	46 (17.8%)	17 (18.3%)	
Secondary	147 (41.8%)	108 (41.7%)	39 (41.9%)	
Tertiary	139 (39.5%)	104 (40.2%)	35 (37.6%)	
Employment				0.21
Not Employed	121 (34.4%)	94 (36.3%)	27 (29.0%)	
Employed	231 (65.6%)	165 (63.7%)	66 (71.0%)	
Family Planning Method				0.83
Not natural	219 (62.2%)	162 (62.5%)	57 (61.3%)	
Natural	133 (37.8%)	97 (37.5%)	36 (38.7%)	
Trimester				0.11
First	49 (13.9%)	39 (15.1%)	10 (10.8%)	
Second	165 (46.9%)	127 (49.0%)	38 (40.9%)	
Third	138 (39.2%)	93 (35.9%)	45 (48.4%)	
Number of partners				0.46
More than one	2 ( 0.6%)	1 ( 0.4%)	1 ( 1.1%)	
One	350 (99.4%)	258 (99.6%)	92 (98.9%)	
History of STD				0.21
No	175 (49.7%)	134 (51.7%)	41 (44.1%)	
Yes	177 (50.3%)	125 (48.3%)	52 (55.9%)	
History of Diabetes Mellitus				0.57
No	348 (99.1%)	256 (98.8%)	92 (100.0%)	
Yes	3 ( 0.9%)	3 ( 1.2%)	0 ( 0.0%)	
History of *Candida*				0.47
No	208 (59.1%)	156 (60.2%)	52 (55.9%)	
Yes	144 (40.9%)	103 (39.8%)	41 (44.1%)	
History of Antibiotic Use				0.76
No	269 (76.4%)	199 (76.8%)	70 (75.3%)	
Yes	83 (23.6%)	60 (23.2%)	23 (24.7%)	
History of Chronic Condition				0.88
No	323 (91.8%)	238 (91.9%)	85 (91.4%)	
Yes	29 ( 8.2%)	21 ( 8.1%)	8 ( 8.6%)	
Para				0.10
0	90 (25.7%)	72 (28.0%)	18 (19.4%)	
1	260 (74.3%)	185 (72.0%)	75 (80.6%)	

Prevalence of candidiasis among pregnant women

**Figure 1:**
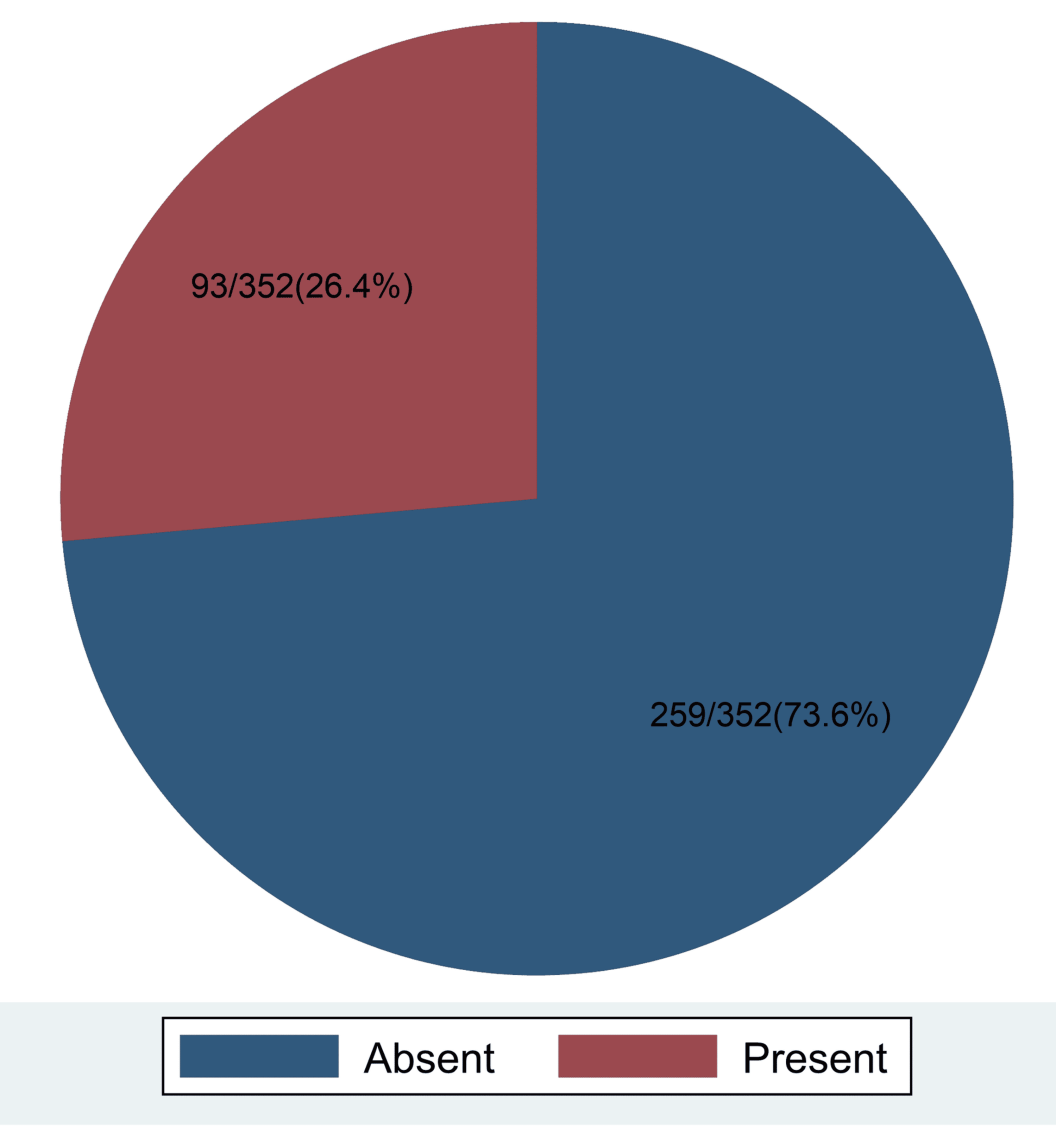
Prevalence of Candidiasis among Pregnant Women

A total of 352 respondents were screened for vulvovaginal candidiasis. Out of this total, 93 were diagnosed with VVC, giving an overall prevalence of 26.4% with a 95% CI of 22.1% to 31.3%. The results are shown in Figure 1.

Common *Candida* species causing VVC

**Figure 2:**
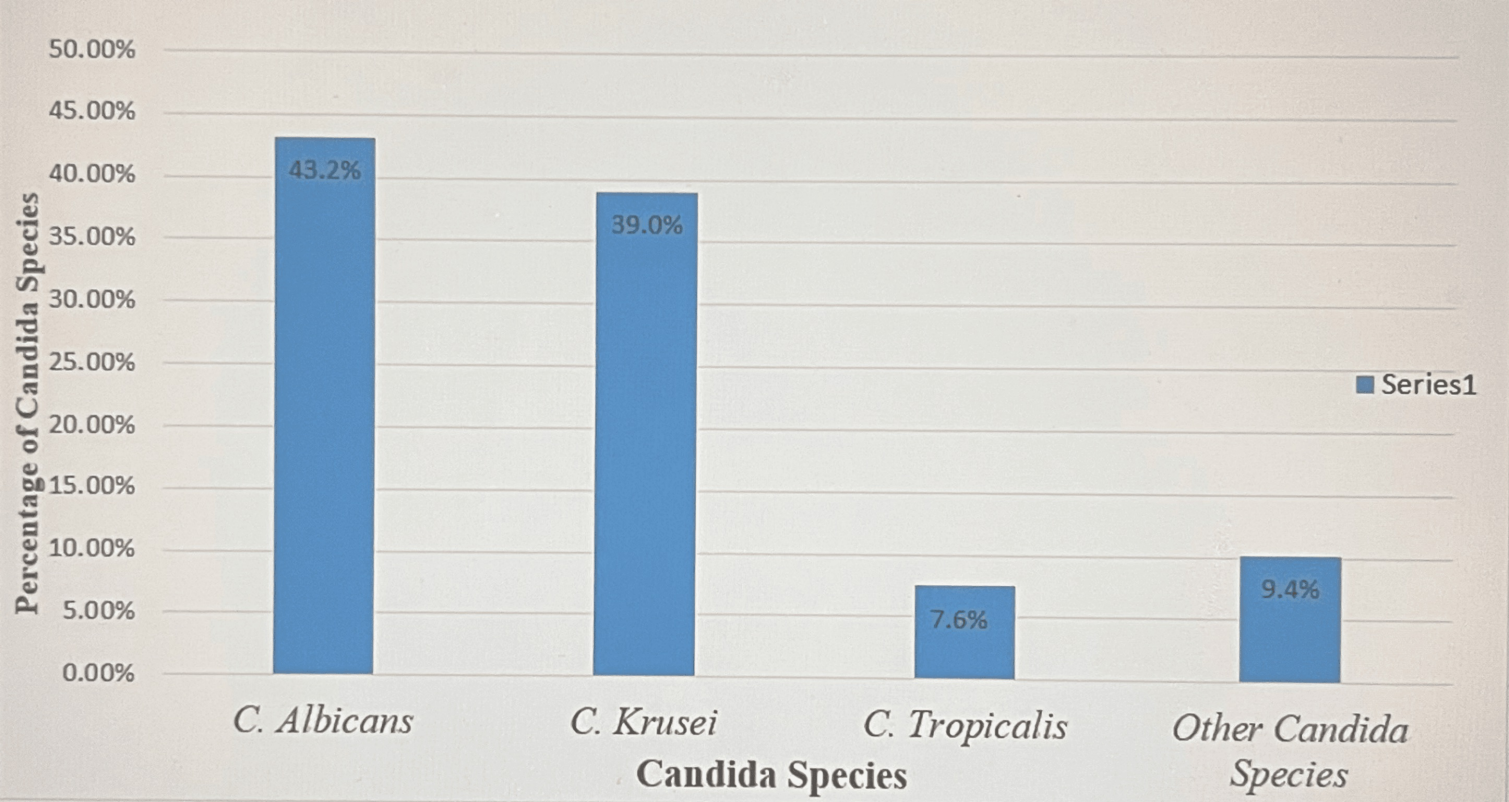
Common Candida Species causing Vulvovaginal Candidiasis

In this study, *C. albicans* (n=50, 42.4%) and *C. krusei* (n=45, 38.1%) were the most common species, and the least common species were *C. tropicalis* (n=9, 7.6%) and other *Candida* species (n=14, 11.9%), as shown in Figure 2. 

Drug susceptibility patterns of common *Candida* species 

**Figure 3:**
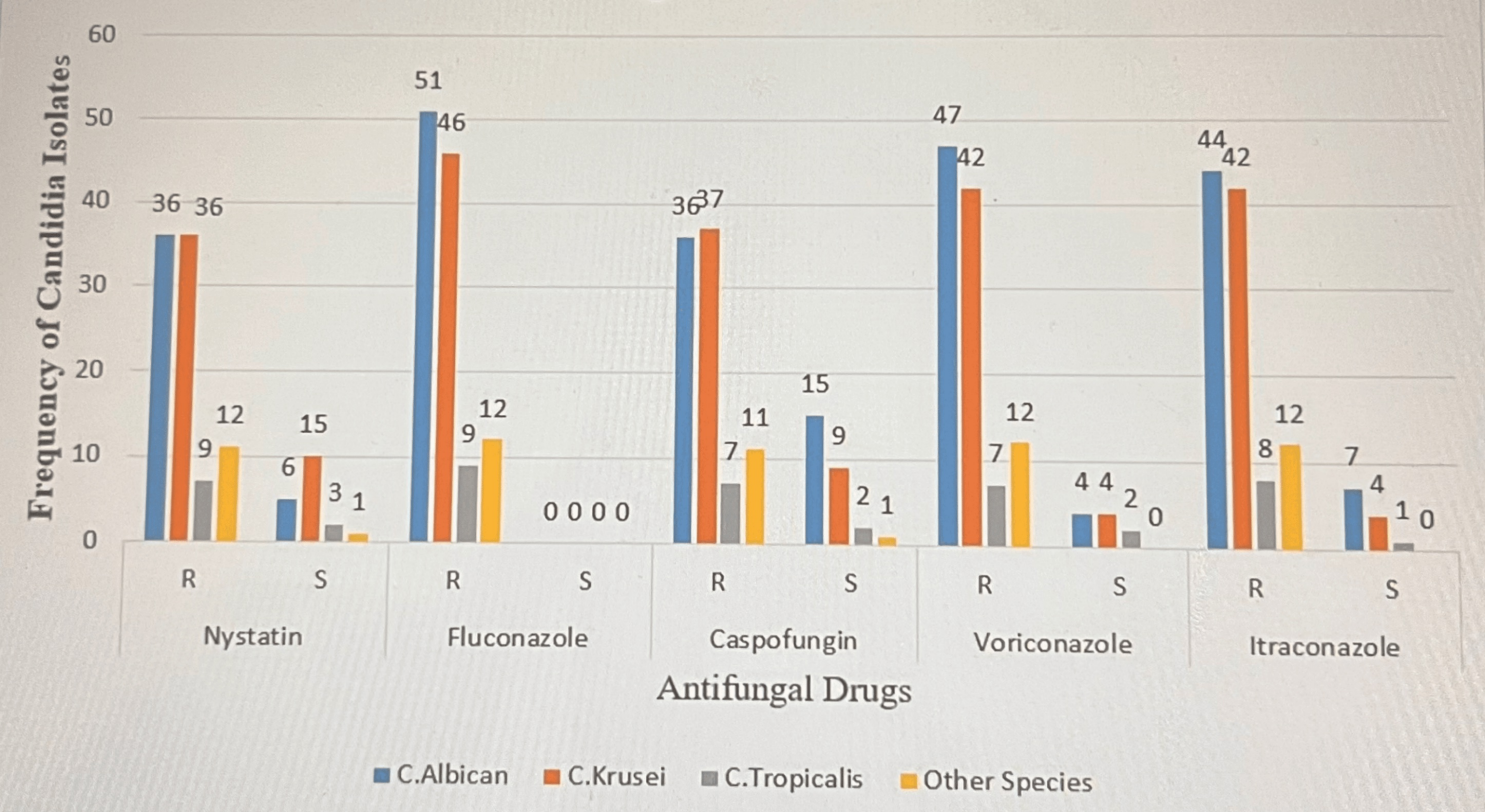
Drug Susceptibility Pattern of Most Candida Species R: resistant; S: sensitive

Total resistance to all the antifungal agents used was rated at 86.9% and a multidrug resistance of 56.3% was expressed by the *Candida* isolates. Up to 100% of the *Candida* species were resistant to fluconazole (100%) and least resistant to nystatin at 76.3%. *C. albicans* expressed the highest resistance, which was rated as follows with the antifungal agents used: fluconazole 100 μg (n=36, 39.8%), itraconazole 10 μg (n=42, 35.6%), voriconazole 1 μg (n=42, 35.6%), caspofungin 5 μg (n=37, 31.4%), and nystatin 100 μg (n=36, 30.5%). The drug susceptibility patterns of the common *Candida* species are shown in Figure 3.

## Discussion

Globally, *Candida* is the most common opportunistic fungal infection and is responsible for 90% of the cases of infectious vaginitis [10]. This cross-sectional study was carried out because the diagnosis and treatment of VVC are normally done without any laboratory antifungal susceptibility testing. A total of 352 participants took part in the study, of whom the overall prevalence of VVC was 26.3%. This finding is close to studies done by Nurat et al. at Lautech Teaching Hospital in Ogbomoso, Nigeria [11], Tsega and Mekonnen at Debre Markos Referral Hospital, Northwest Ethiopia [12], and Yasin et al. in Gondar, Northwest Ethiopia [13], which reported prevalences of 25%, 25%, and 23.8%, respectively. This could be attributed to the use of similar laboratory diagnostic techniques and similarities in study population and settings. In other studies, a higher prevalence compared to ours was reported. For instance, Yadav and Prakash reported 35% in 2016 [14], Okonkwo and Umeanaeto reported 30% [15], and Waikhom et al. reported 30.7% [16]. This variation in the prevalence across a number of studies could be due to variations in sample size used, laboratory diagnostic methodology, and the gestation condition of the participants.

This study found the highest numbers of VVC in the age group of 21-29 years (52.6%), followed by the age group of 30-39 years with 33.8%. This is similar to a study by Nurat et al. [11] and Yadav and Prakash [14]. The occurrence of higher cases of VVC in the age group of 21-29 years could be attributed to them being young and more sexually active compared to those above 40 years, who may have decreased levels of oestrogens, a pregnancy hormone which reduces the ability of vaginal epithelial cells to inhibit the growth of *C. albicans* [17]. The physiological and tissue changes, due to reproductive hormones, which usually occur in young women, especially during pregnancy, increase their susceptibility to *Candida* infection [18]. A study by Mushi et al. [17] supports this explanation of changes in sex hormones leading to increasing chances of *Candida *vaginitis. 

This study found *C. albicans* (43.2%) as the predominant organism causing VVC in pregnancy, followed by *C. krusei* (38.9%). These results corroborate that of Nurat et al. [11] and Yadav and Prakash [14]. However, in a study done in Ghana by Waikhom et al., *C. glabarata* was reported as the most predominant organism, followed by *C. albicans *[16]. This disagreement of the findings could be a result of self-medication and prolonged antifungal therapy, which may have led to the selection of *C. glabrata* over *C. albicans*, due to their increased resistance to the commonly used antifungal agents that can be purchased over the counter [19]. The variation in the aetiology could be due to variations in the environmental, ethnic, socio-economic, and cultural factors, as previously observed in the study by Toua et al. [18].

The current study has shown that 100% of *Candida* isolates were resistant to fluconazole by the diffusion method. This was far above the documented percentage in other studies in Ghana (48.1%) [16], India (19.44%) [20], and Nigeria (36.2%) [21]. This high resistance to fluconazole is likely due to the extensive use of fluconazole as empiric treatment of VVC in our settings, which could have resulted in selection pressure, as previously reported by Pam et al. in Nigeria [22]. Unlike *C. krusei*, known to have intrinsic resistance to fluconazole, resistance expressed by *C. albicans* may have been acquired. Additionally, widespread use of azole antifungals has been associated with the emergence of resistant *Candida *species, notably *C. krusei, *especially in immunosuppressed individuals who may have recurrent infections and hence long-term use of the antifungals.

This study showed that *Candida *species had decreased resistance to the polyenes (nystatin) compared to the azoles (fluconazole and voriconazole) and echinocandin (caspofungin). This is consistent with previous reports that suggested that yeasts are universally susceptible to polyenes [23]. Additionally, previous studies have reported that echinocandin resistance may be associated with cross-resistance to azole antifungals [24], and so, probably the increase in the azole resistance that is reported in our study may be due to cross-resistance.

In the present study, 56.3% of the *Candida* species that exhibited resistance to three or more antifungal drugs are categorized as multidrug-resistant (MDR) fungal species. Except for nystatin (Polyene), *Candida* species showed high resistance to all azoles and echinocandins (caspofungin) tested. This is not consistent with the findings by Dhasarathan et al. [24], which reported 85.3% MDR *Candida *species. This could be due to the difference in study population characteristics and the classes of drugs used. Contrary to previous studies, which have shown multidrug resistance to the azoles, echinocandins, and polyenes to be common among NDAs, notably in *C. glabrata *and *Candida auris* [25], our study reports multidrug resistance among *C. albicans*. This is worrying since* C. albicans* is a common cause of vaginitis and invasive candidiasis, and previously, antifungal resistance was reported to be less common in *C. albicans*. The occurrence of multidrug resistance among *C. albicans* could be due to long-term use of antifungals, unnecessary use of antifungals, or self-medication, which is a common practice among people living in resource-limited settings.

Limitations

This study was conducted at a single peri-urban hospital, which may limit the generalizability of the findings to other regions or healthcare settings in Uganda. Laboratory identification relied on conventional culture and biochemical methods without molecular confirmation, which may have limited the accuracy in differentiating closely related *Candida* species. The 100% fluconazole resistance of all *Candida* species should be examined critically through larger multicenter studies.

## Conclusions

*C. albicans* and *C. krusei* remain significant causes of candidiasis among pregnant women in our setting. The observed multidrug resistance in *C. albicans* poses a great threat to the management of candidiasis. Strengthening antifungal stewardship programs and updating local treatment guidelines are essential to address emerging resistance. Further large-scale studies are also needed to monitor evolving resistance in *Candida* species.
